# High-Fat Diet Enhances Platelet Activation and Is Associated with Proprotein Convertase Subtilisin Kexin 9: An Animal Study

**DOI:** 10.3390/nu15204463

**Published:** 2023-10-21

**Authors:** Fadlina Chany Saputri, Nuriza Ulul Azmi, Meidi Utami Puteri, Damayanti, Vivi Novita, Gracia Marisi, Elin Oktavira, Aninda Novika Sari, Khairunisa Ronaningtyas, Enny Herawati

**Affiliations:** 1 Laboratory of Pharmacology-Toxicology, Faculty of Pharmacy, Universitas Indonesia, Kampus UI, Depok 16424, West Java, Indonesia; nuriza.azmi@farmasi.ui.ac.id (N.U.A.); meidiutami@farmasi.ui.ac.id (M.U.P.); aninda.novika@ui.ac.id (A.N.S.); khairunisa.ronaningtyas@ui.ac.id (K.R.); enny.herawati@ui.ac.id (E.H.); 2 National Metabolomics Collaborative Research Center, Faculty of Pharmacy, Universitas Indonesia, Kampus UI, Depok 16424, West Java, Indonesia; 3 Laboratory of Drug Development, Faculty of Pharmacy, Universitas Indonesia, Kampus UI, Depok 16424, West Java, Indonesia; damayanti72@ui.ac.id (D.); vivi.novita71@ui.ac.id (V.N.); gracia.marisi@ui.ac.id (G.M.); elin.oktavira@ui.ac.id (E.O.)

**Keywords:** hyperlipidemia, hypercholesterolemia, hypertriglyceridemia, high-fat diet, PCSK9, platelet activation

## Abstract

Platelet activation and proprotein convertase subtilisin kexin 9 (PCSK9) play pivotal roles in the progression of atherosclerosis to cardiovascular events. It has been reported that hyperlipidemia, a well-documented risk factors for cardiovascular diseases, tends increase platelet activation and PCSK9 expression. However, little is known about this specific mechanism, particularly how nutrition affects platelet activation and PCSK9 levels in hyperlipidemia conditions. This study aimed to assess how a high-fat diet influences platelet activation, its association with PCSK9, and the effects on blood pressure in an animal model. Here, male Wistar rats were divided into four groups, subjected to different high-fat diets for ten weeks with varying nutrient components. The results showed that high-fat diet-induced hypercholesterolemia and hypertriglyceridemia significantly increased the plasma levels of β-thromboglobulin (β-TG), *p*-selectin, and platelet factor 4 (PF-4). The blood pressure readings were also elevated post high-fat diet induction. Interestingly, the group with the highest percentage of saturated fatty acid and trans-fat exhibited the highest PCSK9 levels, along with the highest increase in plasma cholesterol, triglycerides, and platelet activation parameters. These findings confirm that high-fat diet-induced hypercholesterolemia and hypertriglyceridemia stimulate platelet activity and PCSK9 levels. Moreover, our results suggest that PCSK9, implicated in hypercholesterolemia and hypertriglyceridemia, may synergistically mediate platelet hyperactivity, aligning with clinical studies. Notably, our results highlight the association between a high-fat diet and PCSK9, providing insights for drug discovery targeting platelet activation in atherosclerosis-induced cardiovascular diseases.

## 1. Introduction

Platelets play an important role in the process of thrombus formation in homeostasis to prevent bleeding and in the pathogenesis of cardiovascular diseases [1]. Platelet dysfunction contributes to the initiation and aggravation of atherosclerosis, resulting in cardiovascular diseases, including myocardial infarction and strokes [2]. The pathophysiology of myocardial infarction involves platelet activation leading to the production of plaque that promotes thrombotic occlusion of the coronary vessels, causing ischemia due to reduced blood flow [3]. Myocardial infarction may result in premature death, and until recently, cardiovascular disease remained a global health problem, with estimates showing it to be the leading cause of death worldwide [4]. Dyslipidemia, hypertension, smoking, and diabetes are among the well-known modifiable risk factors of myocardial infarction [5].

Dyslipidemia, a lipid metabolism disorder, is one of the risk factors for atherosclerotic and thrombotic events, leading to atherosclerotic cardiovascular disease, a major cause of death among patients with dyslipidemia [6]. In dyslipidemia, the progression of atherosclerosis and thrombotic events involve platelet hyperactivation, indicating that dyslipidemia may hyperactivate the platelets, which subsequently aggravate atherothrombosis [6,7]. Hypercholesterolemia may cause atherothrombotic events by promoting platelet production and activation [8]. A previous clinical study by Pawelczyk et al. showed that hyperlipidemia exerted a stimulatory effect on *p*-selectin, a biomarker of platelet activation [1]. Another study reported that patients with hypertriglyceridemia exhibited increased platelet activation [9]. Activated platelets release numerous proteins, such as *p*-selectin, beta-thromboglobulin (β-TG), platelet factor 4 (PF-4), thromboxane A2 (TxA2), RANTES, CD40L, and several other molecules, which can potentially be used as biomarkers for cardiovascular disease [10,11]

Proprotein convertase subtilisin kexin 9 (PCSK9) has attracted attention as a therapeutic target for patients with hypercholesterolemia due to its ability to reduce low-density lipoprotein cholesterol (LDL-C) levels [12]. Playing a crucial role in cholesterol homeostasis, PCSK9 promotes the degradation of LDL receptors, leading to increased LDL-C; thus, inhibiting PCSK9 results in decreased LDL-C [12]. Recent research has brought to light additional roles of PCSK9 in cardiovascular events beyond its regulation of LDL-C [13]. These roles include its involvement in stimulating platelet activation during the progression of cardiovascular diseases [13]. Consequently, PCSK9 appears as a promising target for preventing and treating cardiovascular incidents.

The development of PCSK9 inhibitors has strengthened this prospect, as demonstrated by their ability to promote cardiac function in a rat model of acute myocardial infarction (AMI) [14]. Clinical studies have noted elevated concentrations of PCSK9 in patients with hypercholesterolemia [15]. Furthermore, several studies have established a connection between PCSK9 levels and myocardial infarction patients, as well as high-risk cardiovascular patients, such as those with hypercholesterolemia and/or diabetes [15,16,17]. PCSK9’s role in regulating platelet activation by enhancing platelet reactivity has also been documented [18,19,20]. Despite these valuable insights, the study of how nutrition or diet intake influences lipid profile, PCSK9, and platelet reactivity leading to cardiovascular events remains significantly limited.

To the best of our knowledge, there has not been a study on the impact of hypercholesterolemia and hypertriglyceridemia on platelet activity and PCSK9 using animal models. Recognizing the significance of these research gaps, the present study is designed to investigate the correlation between induced hypercholesterolemia and hypertriglyceridemia resulting from a high-fat diet and the manifestation of platelet activation biomarkers in rat models. The development of pathological conditions in an animal study may be useful in developing new drug targets or new therapeutic agents in preclinical studies. Therefore, the current study aimed to investigate the correlation between high-fat diet-induced hypercholesterolemia and hypertriglyceridemia and platelet activation biomarkers in rats, as well as to determine the involvement of PCSK9 expression in modulating platelet reactivity in high-fat diet conditions.

## 2. Materials and Methods

### 2.1. Animals and High-Fat Diet

**Figure 1:**
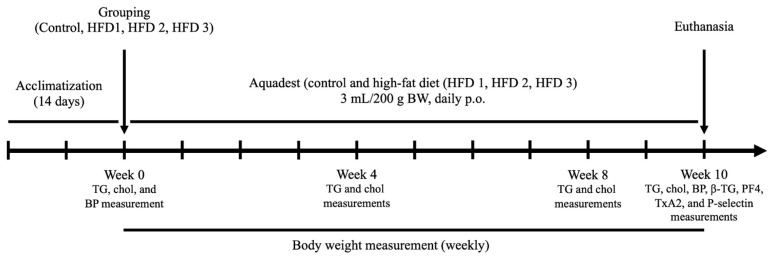
Design of the animal study for 10 weeks with male Wistar rats. TG: triglyceride; chol: cholesterol; BP: blood pressure; β-TG: beta-thromboglobulin; PF4: platelet factor 4.

All animals used in this experiment were 3-month-old male Wistar rats weighing 200–300 g and purchased from U.D Wistar Yogyakarta, Indonesia. Prior to the experiments, all rats were acclimatized for 14 days in the animal house of the Laboratory of Pharmacology and Toxicology, Faculty of Pharmacy, Universitas Indonesia. The conditions were kept at room temperature with a 12 h light and dark cycle. After acclimatization, the rats were randomly divided into the following four groups (*n* = 5/group): control group, high-fat diet 1 group (HFD 1), high-fat diet 2 group (HFD 2), and high-fat diet 3 group (HFD 3). The design of the animal study is shown in Figure 1.

The study was performed in accordance with the Animal Act of 1986 guideline and approved by the Ethics Committee of the Faculty of Medicine, Universitas Indonesia (reference number KET-45/UN2.F1/ETIK/PPM.00.02/2021). All experimental groups were fed daily with a high-fat diet orally for ten weeks, whereas the control group received aquadest. The high-fat diet model utilized in this study was adapted from previous successful studies that induced hyperlipidemia, including hypercholesterolemia and hypertriglyceridemia [21,22,23]. A modification was made in the composition of fat sources (quail egg yolk, goat fat, beef fat, and butter) to vary the nutrient components, mimicking dietary diversity. The compositions of the high-fat diets provided to HFD 1, HFD 2, and HFD 3 are displayed in Table 1. The nutrition and calories status are showed in Table 2 and Table 3, respectively. To manage the administration of HFD induction, we utilized oral gavage once a day every morning at 7 a.m. for a duration of ten weeks constitutively. During this time, the animals had unrestricted access to regular food and water, with the exception of the day when blood samples were collected. On that specific day, they underwent a fasting period of 10–12 h prior to the blood collection. Body weight was measured weekly for ten weeks. The nutrition and caloric status of regular food are displayed in Supplementary Table S1.

### 2.2. Triglyceride and Cholesterol Measurement

Commercial kits for total cholesterol (Human, Germany) and triglyceride (Human, Germany) were used to measure plasma triglycerides and cholesterol at weeks 0, 4, 8, and 10 following the manufacturer’s instructions. Absorbance was obtained at a wavelength measurement of 500 nm and converted into mg/dL to determine the plasma concentration.

### 2.3. Systolic, Diastolic, and Arterial Blood Pressure Measurement

The systolic, diastolic, and arterial blood pressures were determined before and after induction of a high-fat diet using a non-invasive CODA (Kent Scientific Corporation, Torrington, CT, USA) based on the methods performed by a previous study [30]. Before the measurement, all rats were moved to a chamber and allowed to acclimatize for 15–30 min at 30 °C.

### 2.4. Blood Collection

Rats underwent a fasting period of 10–12 h prior to blood collection. At weeks 4, 8, and 10, fasting occurred after the administration of a high-fat diet (HFD), starting around 8 a.m., with blood collection taking place 10–12 h later, approximately between 6 and 8 p.m. Blood samples from the orbital sinus were obtained at weeks 0, 4, and 8 to measure the plasma triglyceride and cholesterol levels. The collected volume did not exceed 1.5% of the rat’s weight; for instance, for a rat weighing 250 g, the maximum volume would be 3 mL. Throughout the 10-week induction period, all rats were anesthetized intraperitoneally using ketamine at a dose of 100 mg/kg body weight. Blood collection was conducted via the abdominal aorta, with a blood volume of 6–8 mL, following the method outlined by Gage et al., with slight modifications [31]. To prevent hemolysis, blood collection was performed with care. Heparin-containing blood was then centrifuged at 3000 rpm for 10 min, and the obtained plasma was stored at −20 °C for further measurements.

### 2.5. Biochemical Parameters for Platelet Activation and PCSK9

The plasma levels of the platelet activation parameters and PCSK9 were measured using enzyme-linked immunosorbent assay (ELISA) kits following the manufacturer’s instructions. All platelet activation parameters were analyzed using rat ELISA kits for β-TG, PF4, TxA2, and *p*-selectin purchased from Cloud-Clone (Cloud-Clone Corp, Houston, TX, USA). Meanwhile, the rat ELISA kit for PCSK9 was obtained from Cusabio (Cusabio Biorech Co, Ltd., Wuhan, China.) The wavelength measured using the Glomax^®^ multi detection system (Promega, Madison, WI, USA) was 450 nm.

### 2.6. Statistical Analysis

GraphPad Prism 8 was used for statistical analysis. All data are presented as means ± standard deviation (SD) of five rats. Differences were determined using ANOVA, followed by a post hoc test, with *p* < 0.05 indicating statistical significance.

## 3. Results

### 3.1. High-Fat Diet Induces Hypercholesterolemia and Hypertriglyceridemia in Wistar Rats

All groups were fed a high-fat diet orally every day, except for the control group, which received aquadest. As expected, high-fat diet induction in all groups promoted significantly higher plasma cholesterol and triglyceride levels after 10 weeks compared to before induction and the control group (Table 4). Cholesterol levels had increased significantly after 4 weeks of high-fat diet feeding, whereas triglyceride levels increased after 8 weeks in all groups. After 10 weeks of induction, all groups satisfied the conditions for hypercholesterolemia and hypertriglyceridemia, with plasma levels of ≥140 and ≥120 mg/dL, respectively [32].

### 3.2. Body Weight and Blood Pressure Changes following High-Fat Diet Induction

**Figure 2:**
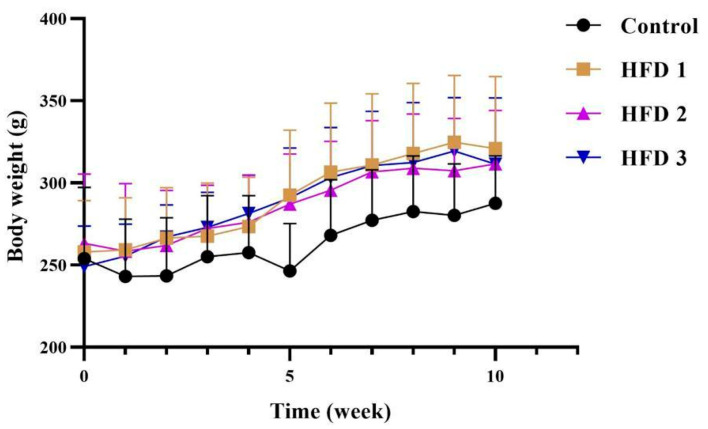
Body weight of rats fed a high-fat diet for 10 weeks. Data are presented as mean ± SD (*n* = 5 rats/group).

**Figure 3:**
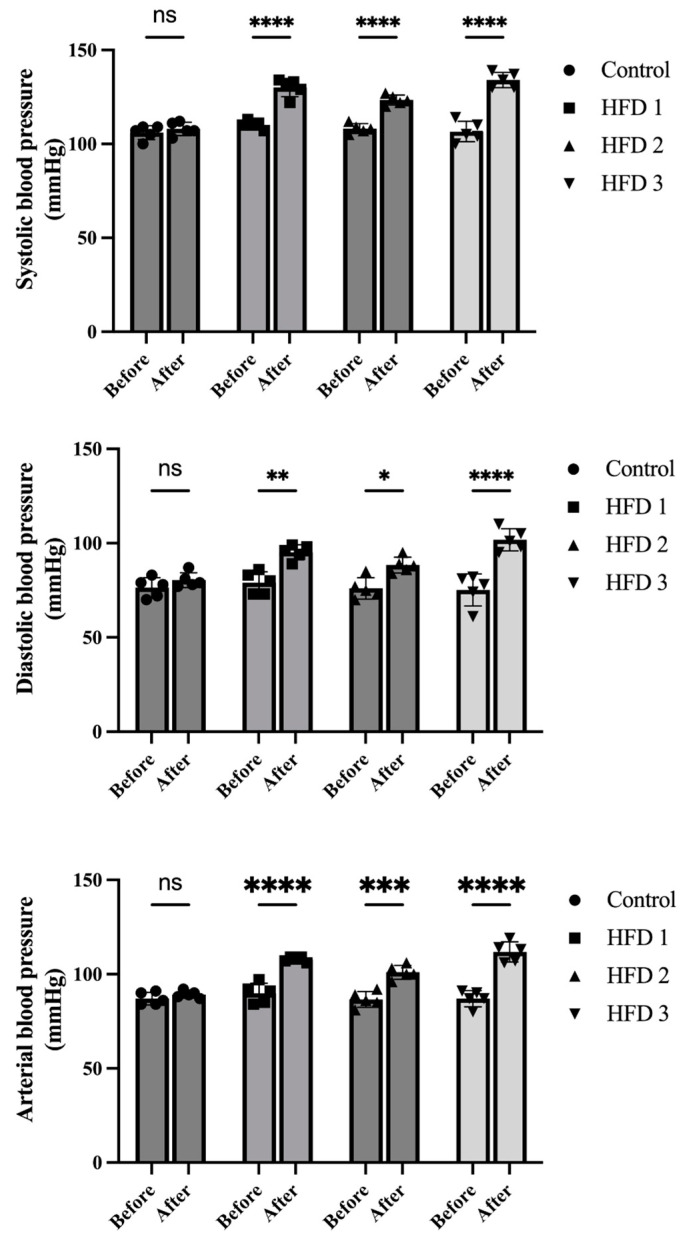
The impact of high-fat diet consumption for 10 weeks on systolic, diastolic, and arterial blood pressures in rats. Data are presented as mean ± SD (*n* = 5 rats/group). Statistical analysis was performed using a 2-way ANOVA, followed by Bonferroni’s multiple comparison test; ns: no significant, * *p* < 0.05, ** *p* < 0.01, and *** *p* < 0.001, **** *p* < 0.0001 denote statistical significance compared to corresponding group before induction.

To evaluate the impact of a high-fat diet in rats, body weight and blood pressure were also evaluated. Body weight, which was measured weekly, showed a stable increase in all groups, with no significant difference compared to the control group (Figure 2). Meanwhile, their systolic, diastolic, and arterial blood pressures, which were also measured before and after 10 weeks of high-fat diet feeding, were higher after than before treatment in the high-fat diet groups (Figure 3).

### 3.3. Hypercholesterolemia and Hypertriglyceridemia Conditions Increase β-TG, p-Selectin, and PF-4 in a Similar Pattern in Wistar Rats

**Figure 4:**
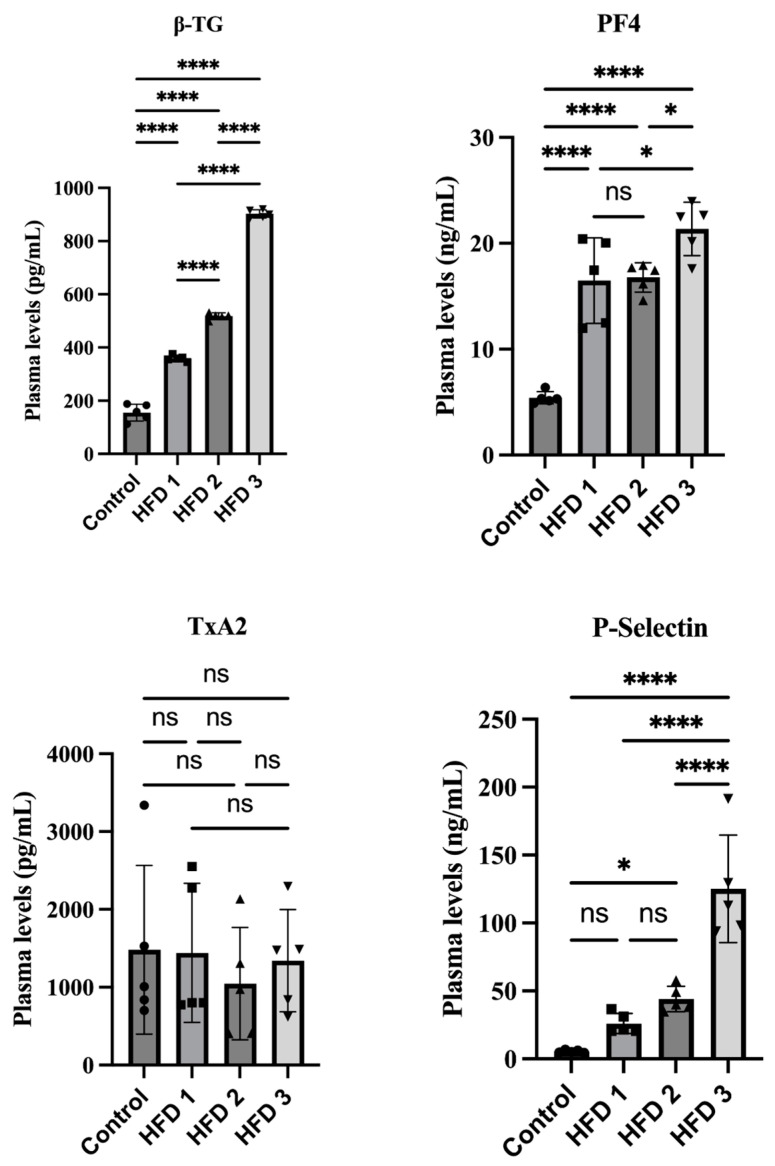
The impact of high-fat diet consumption for 10 weeks on plasma levels of platelet activation parameters in rats. Data are presented as mean ± SD (*n* = 5 rats/group). For β-TG, PF4, and *p*-selectin, statistical analysis were performed using one-way ANOVA, followed by Tukey’s multiple comparison test. For TxA4, statistical analysis were performed using Kruskal–Wallis test, followed by Dunn’s test; ns: no significant, * *p* < 0.05, **** *p* < 0.0001 denote statistical significance compared to control group.

All rats were sacrificed to collect blood plasma after all high-fat diet groups developed hypercholesterolemia and hypertriglyceridemia at week 10. The plasma levels of β-TG, *p*-selectin, PF-4, and TxA2 were analyzed using ELISA to investigate platelet reactivity in our hypercholesterolemia/hypertriglyceridemia rat model. Figure 4 presents the impact of a 10-week high-fat diet on the platelet activation parameters. Accordingly, our results showed that β-TG, *p*-selectin, and PF-4 increased significantly together with cholesterol and triglyceride levels in various high-fat diet groups. Similar to the lipid profile results, HFD 3 exhibited the highest plasma levels of β-TG, *p*-selectin, and PF-4, followed by HFD 2 and HFD 1.

### 3.4. Plasma PCSK9 Levels Were Increased in High-Fat Diet Rats

**Figure 5:**
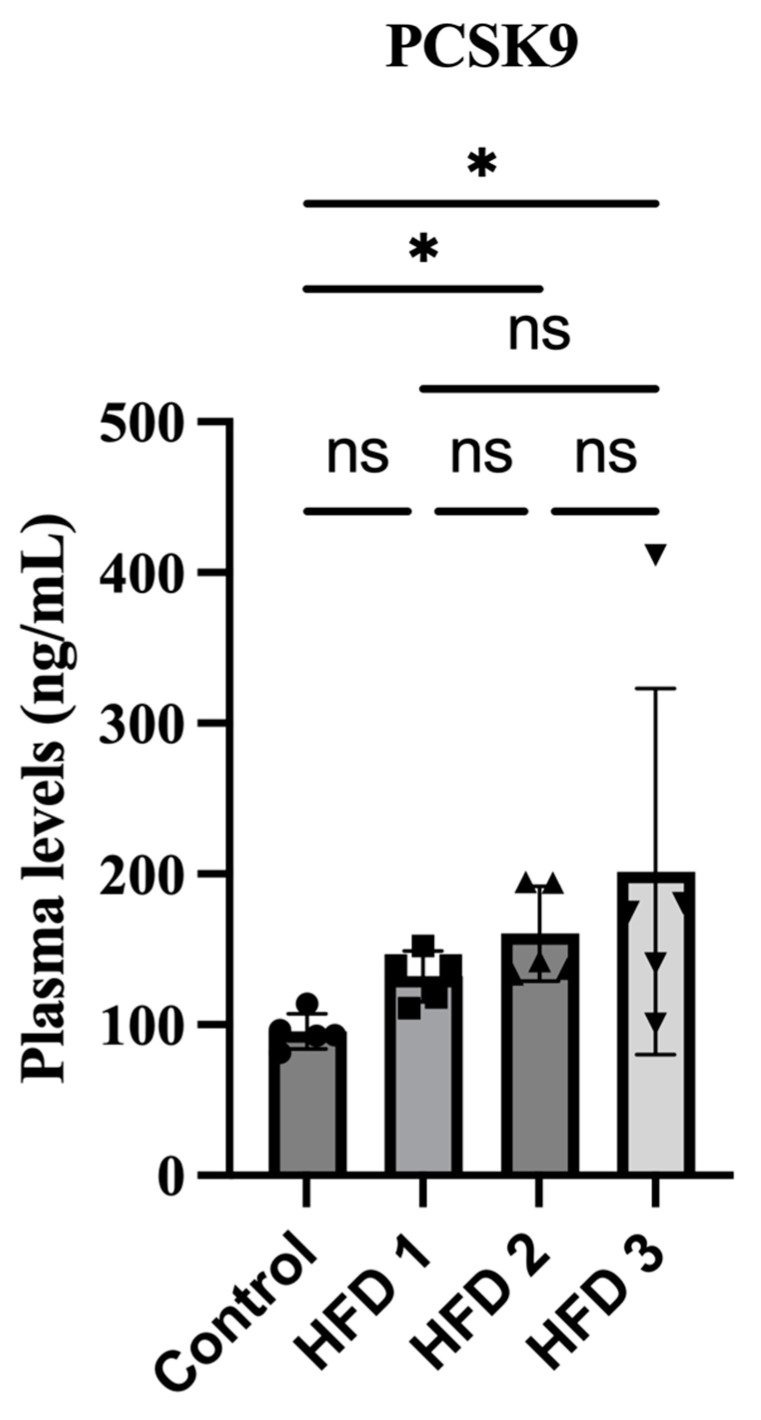
The impact of high-fat diet consumption for 10 weeks on plasma PCSK9 levels in rats. Data are presented as mean ± SD (*n* = 5 rats/group). Statistical analysis was performed using Kruskal–Wallis, followed by Dunn’s test; * *p* < 0.05 denotes statistical significance compared to the control group.

Previous studies have suggested the potential association between platelet activation in the high-fat diet rat model and PCSK9 expression. To further explore this association, the plasma PCSK9 levels were analyzed using ELISA. Our result showed that the plasma levels in HFD 2 and HFD 3 were also significantly increased after 10 weeks of induction together with cholesterol, triglyceride, and the platelet activation parameters (Figure 5). HFD 3 showed the highest PCSK9 levels, followed by HFD 2 and HFD 1.

## 4. Discussions

The present animal study aimed to investigate the impact of a high-fat diet on platelet activation, a key factor in the progression of atherosclerosis. Additionally, our investigations explored the relationship between diverse nutritional intake, PCSK9 levels, and platelet activation in an animal model. The overall goal was to deepen our current understanding of how nutrition or diet intake affects PCSK9 levels, impacting platelet activity and increasing the risk of cardiovascular diseases in an animal model. This established model holds promise for identifying new targets in drug development, particularly those addressing PCSK9-induced platelet hyperreactivity.

In the present study, rats were subjected to a daily high-fat diet induction for a duration of 10 weeks. This diet varied in fat components and total calories, resulting in an increase in cholesterol and triglyceride levels. Interestingly, the rats developed hypercholesterolemia and hypertriglyceridemia in all groups of HFD. This model may help better understand platelet dysfunction in dyslipidemia conditions. Given the important role of diet in the blood levels of lipid and lipoprotein, hyperlipidemia may be influenced by lifestyle [21]. A number of animal studies have established a high-fat diet model for inducing hyperlipidemia, including hypercholesterolemia and hypertriglyceridemia [21,22,23]. It has been well reported that consuming foods high in cholesterol, fructose, saturated fat, and calories increases total cholesterol, triglycerides, and LDL levels, potentially triggering health issues [33]. Indeed, our results showed that various components of the high-fat diet increased cholesterol and triglyceride levels in all groups compared to the control group. Among these, HFD 3, characterized by the highest content of saturated fatty acids and total calories, along with the group containing solely the trans fatty acid component, exhibited the highest values, followed by HFD 2 and HFD 1. Different diets containing high fat levels will result in different levels of profile lipid [34]. Moreover, the effect of a high-fat diet on body weight was also analyzed. A high-fat diet may promote body weight gain given the visceral fat accumulation due to the high fat intake, with the gain varying depending on the intake [35,36]. The current study showed a stable increase in body weight at every week of measurement, with all HFD groups showing a slightly higher body weight compared to the control group, although not significantly.

Dyslipidemia, as well as other factors, such as hypertension, diabetes, metabolic disorders, is a modifiable risk factor for cardiovascular diseases that may be associated with each other [5]. Besides affecting lipid profile, high-fat diet induction may also contribute toward endothelial dysfunction and blood pressure elevation [37]. Consistent with the previous study [38], all HFD groups showed higher systolic, diastolic, and arterial blood pressure values. The high fructose content in the diet may also affect blood pressure by elevating angiotensin II and the expression of the type I angiotensin II (AT1) receptor [39]. Hypertension and hypercholesterolemia have been suggested to be linked through the involvement of the renin–angiotensin system [40]. Both are highly prevalent in the population, and their coexistence increases the risk of cardiovascular disease [40,41,42]

Clinical reports have shown that dyslipidemia can increase platelet activation biomarkers such as *p*-selectin [1,43]. The current study was conducted to confirm the findings of clinical studies on the correlation between hypercholesterolemia/hypertriglyceridemia and platelet activation in rats fed a high-fat diet. All groups that received a high-fat diet exhibited platelet activation through significant elevations in plasma β-TG, *p*-selectin, and PF-4, which may play a role in prothrombotic events. Meanwhile, no significant difference in thromboxane levels were observed. The foreminded results showed a similar pattern with the lipid profile results, suggesting that HFD 3 had the highest plasma levels. In hyperlipidemic conditions, platelets exhibit high reactivity and are more susceptible to activation and aggregation. Therefore, lowering the lipid levels through statins could reduce platelet activity [44]. One study showed that patients who had suffered an ischemic stroke presented platelet hyperactivity, with such a phenomenon potentially associated with hyperlipidemia [45]. By elevating platelet production and directly affecting the platelets themselves, hypercholesterolemia has been found to cause platelet hyperactivation [44]. Activated platelets further form platelet–leukocyte aggregates (PLAs), an independent risk factor of atherothrombotic disease [44,46].

PCSK9 seems to be closely associated with the progression of atherothrombosis, leading to cardiovascular disease, especially in dyslipidemia. Therefore, the current study also investigated alterations in plasma PCSK9 levels during high-fat diet induction. Notably, our results showed that the plasma PCSK9 level increased after 10 weeks of feeding, a finding consistent with that reported by a previous study on the effects of high-fat diet induction on mice for 8 weeks [47] and another study on rats exposed to a long-term high-fat diet [48]. Moreover, dietary fructose appears to upregulate PCSK9 levels through mechanisms related to SREB1c, SREB2, and hepatic LDLR protein [49]. The high-fat diet used in this study contained a component of fructose, which may contribute to the elevation of PCSK9. However, another study showed contrary results, showing no changes in plasma PCSK9 levels after 4 weeks of high-fat diet feeding in rats [50]. The duration of induction may influence the level of PCSK9. After 4 weeks of high-fat diet induction, they showed no significant elevation of total cholesterol. In addition, the LDL-R mRNA expression remained the same. Meanwhile, our study found a significant enhancement of total cholesterol with more than two-fold higher levels, after 10 weeks of high-fat feeding. These results may suggest a strong correlation between the states of hypercholesterolemia with the level of PCSK9 [51]. The well-explained mechanism of PCSK9 synthesis is regulated by the SREB pathway [52]. SREBP-2 stimulates the sterol-dependent transcription of PCSK9 by binding to the sterol regulatory element [53]. Our study did not measure the expression of LDL-R and SREBP-2. Therefore, further studies are needed to elucidate the mechanism underlying PCSK9 elevation in high-fat diet conditions, such as measuring the LDL-R and SREBP-2.

Plasma PCSK9 levels have been positively associated with LDL cholesterol, total cholesterol, and triglyceride [54]. Furthermore, one study showed that plasma PCSK9 concentrations were correlated with triglycerides in patients with chronic kidney disease [55]. Moreover, another study showed that PCSK9 concentrations were also high in patients with hypercholesterolemia [15]. PCSK9 regulates cholesterol homeostasis by binding to the LDLR and inducing degradation, resulting in LDL-C clearance, which consequently leads to hypercholesterolemia due to the accumulation of LDL-C levels [56]. The elevation of plasma PCSK9 in this study may also contribute to hypercholesterolemia. However, one limitation of the current study is that we did not measure the plasma levels of HDL and LDL. Besides regulating LDL-C, PCSK9 has also been found to influence platelet activation. PCSK9 triggers platelet activation by binding to the CD36 on the platelet and as the binding the platelet CD36 downstream signaling pathways [18].

In addition, our results further provide information that the degree of fatty acid saturation may affect not only body weight gain [57] but also the lipid profile, the platelet activation, and the PCSK9 level. This is evidenced by the greater increases in total cholesterol and triglyceride levels, along with platelet activation biomarkers such as plasma β-TG, *p*-selectin, and PF-4, as well as PCSK9. The HFD 3 group, characterized by the presence of trans fatty acids and the highest levels of saturated fatty acids and calories, exhibited the highest biomarker levels, followed by HFD 2 and HFD 1 in descending order of calories and saturated fatty acid concentration. The saturated fatty acid content in HFD 3 is higher compared to HFD 1 and HFD 2 due to the presence of butter and goat fat, each contributing to 50% of the composition. Each percent of saturated fat consumed from the total daily energy is predicted to increase plasma cholesterol levels by 2.7 mg/dL [58]. The elevated level of plasma cholesterol may then promote dyslipidemia, leading to higher platelet reactivity [13,51]. These findings may correlate with reports stating that foods high in trans fatty acid and saturated fatty acids can elevate total blood cholesterol levels, thereby promoting hypercholesterolemia [59,60].

Collectively, our results suggest that a high-fat diet induces hypercholesterolemia and hypertriglyceridemia, leading to an increase in the risk of cardiovascular diseases such as hypertension. Moreover, the variations in nutrition within the high-fat diet impact lipid profiles, platelet hyperreactivity, and PCSK9 elevation, potentially contributing synergistically to the stimulation of platelet activation-induced cardiovascular events.

One limitation of the current study is that we did not measure HDL and LDL concentrations, which could provide information regarding the involvement of PCSK9 and the atherogenic index. We should also consider changes in body weight following a high-fat diet given that the current study showed no significant differences therein. The different components of a high-fat diet and duration of induction may influence the results. Nonetheless, our findings may pave the way for further research aimed at elucidating the correlation between nutrition intake, dyslipidemia, PCSK9, and platelet activation in the pathophysiology of atherosclerosis leading to myocardial infarction [61]. Our results are consistent with the findings of clinical studies, indicating that a high-fat diet resulting in hypercholesterolemia and hypertriglyceridemia is associated with a higher risk of platelet activation and elevated PCSK9 levels. Importantly, the current study established an animal model for investigating PCSK9 and platelet activation following variations in nutrition within a high-fat diet. This animal model may contribute to future studies aiming to elucidate the mechanisms underlying the association among risk factors for myocardial infarction involving PCSK9 and platelet activation.

## 5. Conclusions

Our findings confirmed that hypertriglyceridemia and hypercholesterolemia in male Wistar rats fed a high-fat diet enhanced platelet activation, which is consistent with reports from clinical studies. Additionally, the induction of high-fat diet also increased systolic, diastolic, and arterial blood pressures. Similar to cholesterol, triglycerides, and platelet activation, higher PCSK9 expression levels were observed, suggesting that a high-fat diet may increase PCSK9 levels and that both PCSK9 and hypercholesterolemia/hypertriglyceridemia synergistically modulate platelet hyperactivity. Our results significantly contribute to unraveling the specific mechanisms that correlate with risk factors for cardiovascular diseases, such as myocardial infarction. Notably, according to our findings, a high intake of saturated fatty acids and trans fatty acid from butter appears to substantially elevate the risk of dyslipidemia, platelet reactivity, and increased levels of PCSK9. Furthermore, our studies provide guidance for future studies aiming to elucidate the molecular mechanisms involved in the pathogenesis of platelet activation and atherosclerosis. These mechanisms encompass nutrition intake, PCSK9 expression, and platelet reactivity, offering valuable insights for a deeper understanding of the interplay among these factors. Hence, the established animal study can be useful for drug discovery and development research targeting platelet activation in atherosclerosis-induced cardiovascular disease.

## Figures and Tables

**Table 1:** Compositions of the high-fat diets.

Ingredients	HFD 1 (*w*/*v*)	HFD 2 (*w*/*v*)	HFD 3 (*w*/*v*)
Quail egg yolk	40%	30%	-
Goat fat	25%	-	50%
Beef fat	-	35%	-
Butter	-	-	15%
Cholesterol (Dyets, Bethlehem)	2%	2%	2%
Cholic acid (Wako, Japan)	0.5%	0.5%	0.5%
Fructose	20%	20%	20%
Coconut oil	12.5%	12.5%	12.5%

**Table 2:** Calculation of nutritional status in each ingredient of the high-fat diets. SFA = Saturated Fatty Acids, MUFA = Monounsaturated Fatty Acids, PUFA = Polyunsaturated Fatty Acids, TFA = Trans Fatty Acids. ^#^ nutrition information from packaging.

Ingredients	Weight	Carbohydrates	Protein	Fat (%)				Cholesterol
	(g)	(%)	(%)	SFA	MUFA	PUFA	TFA	(%)
Quail egg yolk	100 [24]	0.20–1.00 [24]	15.70–16.60 [24]	29.90 ± 3.54 [25]	45.02 ± 5.16 [25]	25.09 ± 6.58 [25]	N/A	2.14 [24]
Goat Fat	100 [26]	N/A	N/A	47.32 [27]	40.59 [27]	7.80 [27]	N/A	0.10 [27]
Beef fat	100 [26]	N/A	N/A	49.76 [27]	41.80 [27]	4 [27]	N/A	0.11 [27]
Butter	15 [27] ^#^	0 [27] ^#^	0 [27] ^#^	53.33 [27] ^#^	16.67 [27] ^#^	0 ^#^	3.33 [27] ^#^	0.20 ^#^
Fructose	10 ^#^	80 ^#^	0 [27] ^#^	0 ^#^	0 ^#^	0 ^#^	N/A	N/A
Cholesterol	500 ^#^	N/A	N/A	N/A	N/A	N/A	N/A	100 ^#^
Cholic acid	100 ^#^	N/A	N/A	N/A	N/A	N/A	N/A	N/A
Coconut oil	14 ^#^	0 [27] ^#^	0 [27] ^#^	78.57 ^#^	7.14 ^#^	0 ^#^	0 ^#^	0 [27] ^#^

**Table 3:** Calculation of total calories of each HFD. Weight of high-fat diet (g) = amount of material used × (250 g BW/200 g × 3 mL) × density (g/mL). Calories = weight of high-fat diet (g)/total weight of serving size (g) × total calories in serving size (kcal). Density of butter = 0.911 g/mL [28]; cholesterol = 1.067 g/mL (Merck, Germany); cholic acid = 1.2 ± 0.1 g/mL [29]; egg yolk = 1.03 g/mL [28]; animal fat = 0.7 g/mL [28]; fructose = 1.59 g/mL (Merck, Germany); and coconut oil = 0.903 g/mL [28].

Ingredients	HFD 1		HFD 2		HFD 3	
	Weight (g)	Calories (kcal)	Weight (g)	Calories (kcal)	Weight (g)	Calories (kcal)
Quail egg yolk	1.55	4.99	1.16	3.74	-	-
Goat fat	0.66	5.95	-	-	1.31	11.82
Beef fat	-	-	0.92	8.30	-	-
Butter	-	-	-	-	0.51	3.74
Fructose	1.19	4.17	1.19	4.17	1.19	4.17
Cholesterol	0.08	N/A	0.08	N/A	0.08	N/A
Cholic acid	0.02	N/A	0.02	N/A	0.02	N/A
Coconut oil	0.42	3.30	0.42	3.30	0.42	3.30
Total calories (kcal)		18.41	19.51		23.03	

**Table 4:** Plasma cholesterol and triglyceride changes in rats fed a high-fat diet for ten weeks. Data are presented as mean ± SD (n = 5 rats/group); statistical analyses were performed using one-way ANOVA, followed by Tukey’s multiple comparison test; * *p* < 0.05, ** *p* < 0.01, and *** *p* < 0.001 denote statistically significant compared to corresponding group at week 0; ^#^ *p* < 0.05, ^##^ *p* < 0.01, and ^###^ *p* < 0.001 denote statistical significance compared to corresponding control group.

		Control	HFD 1	HFD 2	HFD 3
Plasma cholesterol (mg/dL)	Week 0	61.22 ± 8.31	60.62 ± 2.88	55.27 ± 6.46	69.70 ± 8.56
	Week 4	68.2 ± 9.54	97.42 ± 13.5 *^#^	94.59 ± 12.19 **^#^	152.61 ± 11.37 **^###^
	Week 8	68.56 ± 6.39	102.07 ± 14.33 *^#^	133.61 ± 14.75 ***^###^	186.99 ± 14.10 ***^###^
	Week 10	69.74 ± 5.66	139.68 ± 11.88 ***^###^	161.14 ± 14.42 ***^###^	248.80 ± 7.48 ***^###^
Plasma triglyceride (mg/dL)	Week 0	71.20 ± 7.58	68.14 ± 9.33	70.36 ± 10.04	68.34 ± 13.51
	Week 4	68.00 ± 13.37	86.75 ± 11.42	106.94 ± 6.70 *^##^	134.39 ± 14.73 **^###^
	Week 8	72.40 ± 14.99	117.99 ± 9.59 **^##^	133.89 ± 13.27 **^###^	199.22 ± 1.80 ***^###^
	Week 10	79.22 ± 14.03	142.06 ± 11.76 ***^###^	185.94 ± 13.36 ***^###^	236.85 ± 7.53 ***^###^

## Data Availability

The data used to support the findings of this study are available upon reasonable request from the corresponding author.

## Supplementary Materials

The following supporting information can be downloaded at https://www.mdpi.com/article/10.3390/nu15204463/s1. Supplementary Table S1. The nutritional information for the Regular Diet used in this study, specifically, HI-PRO-VITE BR 2-CP-5128 for the animal model.
