# Supplementary material for: High-Fat Diet Enhances Platelet Activation and Is Associated with Proprotein Convertase Subtilisin Kexin 9: An Animal Study
Source: Nutrients. 2023 Oct 21;15(20):4463. doi: 10.3390/nu15204463 (PMC10609754; doi:10.3390/nu15204463)
Supplement: Supplementary file 1 [file nutrients-15-04463-s001.zip › nutrients-2636240-supplementary.pdf]

Supplementary Table S1. The nutritional information for the Regular Diet used in this study, specifically, HI-PRO-VITE BR 2-CP-5128 for the animal model.

Component	Percentage
Water	Max 13%
Protein	19-21%
Fat	Min 5,0%
Fiber	Max 5,0 %
Ash	Max 7,0%
Calcium	Min 0,9%
Phosphor	Min 0,6%
Alfatoxyn	Max 50 ppb
Calories	3000-3100 Kcal/k[illegible]
